# Immune Receptors and Co-receptors in Antiviral Innate Immunity in Plants

**DOI:** 10.3389/fmicb.2016.02139

**Published:** 2017-01-05

**Authors:** Bianca C. Gouveia, Iara P. Calil, João Paulo B. Machado, Anésia A. Santos, Elizabeth P. B. Fontes

**Affiliations:** ^1^Department of Biochemistry and Molecular Biology, BIOAGRO, National Institute of Science and Technology in Plant-Pest Interactions, Universidade Federal de ViçosaViçosa, Brazil; ^2^Department of General Biology, BIOAGRO, National Institute of Science and Technology in Plant-Pest Interactions, Universidade Federal de ViçosaViçosa, Brazil

**Keywords:** resistance genes, receptor NIK1, PAMP-triggered immunity, effector-triggered immunity, antiviral immunity, ETI, PTI, NSP-Interacting kinase 1

## Abstract

Plants respond to pathogens using an innate immune system that is broadly divided into PTI (pathogen-associated molecular pattern- or PAMP-triggered immunity) and ETI (effector-triggered immunity). PTI is activated upon perception of PAMPs, conserved motifs derived from pathogens, by surface membrane-anchored pattern recognition receptors (PRRs). To overcome this first line of defense, pathogens release into plant cells effectors that inhibit PTI and activate effector-triggered susceptibility (ETS). Counteracting this virulence strategy, plant cells synthesize intracellular resistance (R) proteins, which specifically recognize pathogen effectors or avirulence (Avr) factors and activate ETI. These coevolving pathogen virulence strategies and plant resistance mechanisms illustrate evolutionary arms race between pathogen and host, which is integrated into the zigzag model of plant innate immunity. Although antiviral immune concepts have been initially excluded from the zigzag model, recent studies have provided several lines of evidence substantiating the notion that plants deploy the innate immune system to fight viruses in a manner similar to that used for non-viral pathogens. First, most R proteins against viruses so far characterized share structural similarity with antibacterial and antifungal R gene products and elicit typical ETI-based immune responses. Second, virus-derived PAMPs may activate PTI-like responses through immune co-receptors of plant PTI. Finally, and even more compelling, a viral Avr factor that triggers ETI in resistant genotypes has recently been shown to act as a suppressor of PTI, integrating plant viruses into the co-evolutionary model of host-pathogen interactions, the zigzag model. In this review, we summarize these important progresses, focusing on the potential significance of antiviral immune receptors and co-receptors in plant antiviral innate immunity. In light of the innate immune system, we also discuss a newly uncovered layer of antiviral defense that is specific to plant DNA viruses and relies on transmembrane receptor-mediated translational suppression for defense.

## Introduction

**FIGURE 1:**
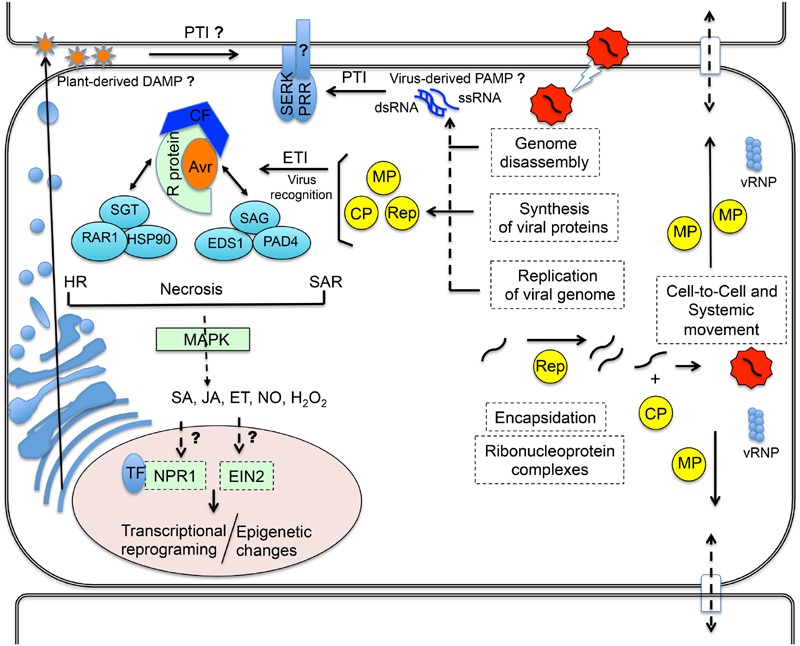
**Antiviral innate immunity with conserved features with antibacterial and antifungal immune responses.** Plant viruses are obligate, biographic parasites and as such their life cycles start with the penetration of the virions in the host cells via wound sites (lightening arrow). Within the host cells, the virion is disassembled and then host cells mediate the expression of the viral genome by providing a translation apparatus for all viruses and transcription machinery for DNA viruses (**Figure 2**). The viral mRNAs are translated into the cytoplasm, producing at least three viral proteins absolutely required for completion of the viral life cycle, replication protein (Rep), movement protein (MP) and coat protein (CP). The viral replication proteins combine with cellular proteins to produce multiple copies of the virus genome. These newly made genomes interact with CPs to form new virions or viral ribonucleoprotein complexes (vRNP). The next step is movement of the virus into neighboring cells, which requires the MP. The intracellular translated viral proteins (Avr) may also provide recognition sites for cytosolic NB-LRR receptors (e.g., R proteins), triggering ETI, which results in HR, necrosis or SAR similarly to non-viral ETI. R proteins, R co-factors (CF) and Avr factors form an interacting complex with the SGT1/RAR1/HSP90, and EDS1/PAD4/SAG101 modules to mediate downstream changes in SA, JA, ET, NO and H_2_O_2_ levels or signaling via MAP Kinases cascades, culminating in the induction of defense genes. NPR1 complexes TF to induce defense genes via SA signaling, whereas EIN2 is a regulator of ET signaling. Virus infection may also trigger epigenetic changes. At the first line of defense, replication of viral RNA genomes may provide non-self RNA motifs (ssRNA or dsRNA) as virus-derived PAMPs to activate PTI. Alternatively, plant cells may sense viral infection and secrete plant-derived DAMPs, recognized by PRRs extracellularly. Members of the SERK family also function as co-receptors in viral PTI. Arrows denote unknown or putative paradigms in viral innate immunity. Adapted from Mandadi and Scholthof (2013).

Plants recognize potential pathogens mainly through two classes of distinct immune receptors (Schwessinger and Ronald, 2012; Spoel and Dong, 2012; Zvereva and Pooggin, 2012; Dangl et al., 2013). The first class consists of cell-surface associated pattern recognition receptors (PRRs), which are often represented by receptor-like kinases (RLKs) and receptor-like proteins (RLPs; **Figure 1**). PRRs recognize conserved structural motifs present in microbes, which are known as microbe- or pathogen-associated molecular patterns (MAMPs/PAMPs), or endogenous danger signals released by the plant during wounding or pathogenic attack, which are termed damage-associated molecular patterns (DAMPs; Macho and Zipfel, 2014). Perception of PAMPs by PRRs activates PAMP-triggered immunity (PTI), a transduction signal cascade that culminates with transcriptional reprograming and biosynthesis of specific defense molecules (Hogenhout et al., 2009; Bigeard et al., 2015). Activation of this immune response enables plants to respond rapidly and efficiently to a large range of pathogens (Roux et al., 2014). The second class of immune receptors includes intracellular immune receptors called R proteins (Jones and Dangl, 2006; Tsuda and Katagiri, 2010; **Figure 1**). These intracellular receptors directly or indirectly recognize effectors secreted by pathogens into the host intracellular environment and activate effector-triggered immunity (ETI; Howden and Huitema, 2012), which is often manifested in the hypersensitive response (HR) associated with rapid cell death, production of reactive oxygen species (ROS) and salicylic acid (SA) as well as expression of defense-related genes (Win et al., 2012). This is considered to be a more robust defense compared to PTI (Coll et al., 2011; Reimer-Michalski and Conrath, 2016). The effectors that are specifically detected by matching R proteins to activate ETI are termed avirulence (Avr) proteins. Pathogens containing Avr genes are avirulent to plants carrying the cognate R genes and virulent to plants without the R genes. Due to the limitation of the coding capacity of viral genomes, virtually all virus proteins, such as replicase, movement proteins (MPs), coat proteins (CPs), can act as Avr determinants. Therefore, virus Avr proteins are usually necessary for successful infection and are almost invariably virulence factors in a susceptible host.

Studies in plant–virus interactions have pioneered the description of paradigms in plant immune response, including the HR and systemic acquired resistance (SAR; Holmes, 1929, 1938; Ross, 1961). Nevertheless, current semantics and concepts regarding plant immunity models were built to fill the findings on bacterial and fungal infections and hence antiviral immune concepts were initially excluded from these models (Jones and Dangl, 2006; Bent and Mackey, 2007; Boller and Felix, 2009; Dodds and Rathjen, 2010; Schwessinger and Ronald, 2012; Spoel and Dong, 2012). Recently, Mandadi and Scholthof (2013) proposed reconciling the differences and perpetuating the analogy between antiviral and anti-non-viral immune concepts into definitions of viral effectors, viral ETI and viral PTI. These definitions, as described below, integrate antiviral immune concepts into current plant immunity models.

Typical bacterial and fungal effectors are delivered into host cells via microbial secretion systems, whereas viral effectors encoded by the viral genome are directly translated into the host cytoplasm. These factors share similar functions because bacterial and fungal effectors interfere with PTI or other immune regulators and viral effectors promote virulence by interfering with host defense pathways. Although not covered in this review, viral suppressors of RNA silencing are also included in this category. Similar to non-viral pathogen effectors, in resistant genotypes, the intracellularly translated viral effectors are recognized by R proteins, triggering immune responses that often are associated with hallmarks of ETI, such as HR, SA accumulation, ROS production and SAR. Therefore, virus Avr factors, which interfere with defenses, are also referred to as viral effectors, and the immune response they trigger is also referred to as ETI. However, viral ETI is independent with regard to the nature of the immune response, which may or may not be associated with hallmarks of bacterial or fungal ETI. The notion that viruses encode PAMPs recognized by PRRs, such as virus-derived nucleic acids, is well documented in animal systems, and recent evidence has extended the concept of viral PTI to plant–virus interaction systems.

**FIGURE 2:**
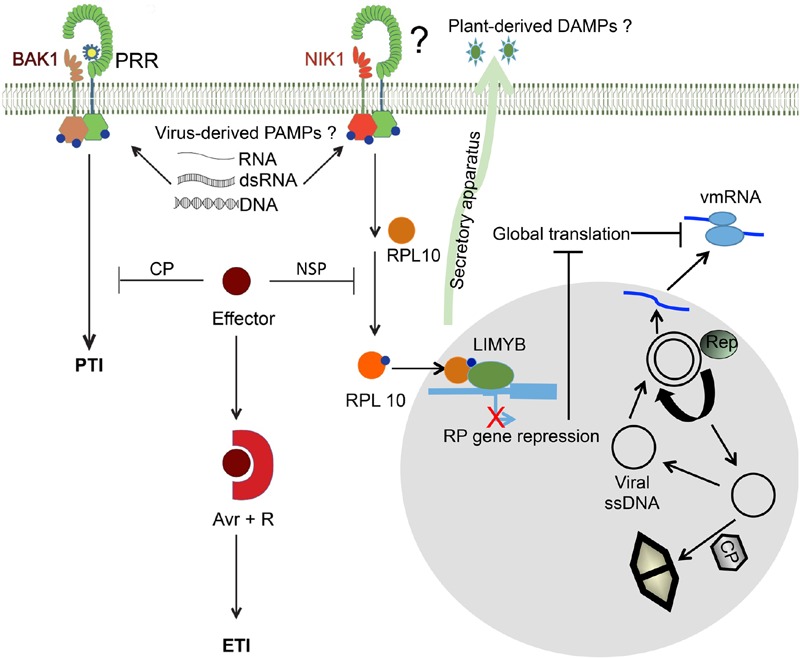
**Similarities between viral PTI and NIK1-mediated antiviral signaling.** Replication and expression of viral genomes lead to the accumulation of non-self DNA or RNA motifs (virus-derived PAMPs), which may be recognized by PRRs that in turn heteromultimerize with co-receptors (BAK1 or SERK1) to trigger viral PTI. Alternatively, PTI may be activated by endogenous DAMPs, which are induced by virus infection and delivered to the apoplast via the secretory apparatus. In addition to PTI, in the case of DNA viruses (begomoviruses), plant cells may also elicit the translational control branch of the NIK1-mediated antiviral signaling as an innate defense. The mechanism of NIK1 transmembrane receptor activation is unknown. Structural organization and biochemical properties of NIK1 may suggest an activation mechanism dependent on recognition of viral PAMPs or endogenous DAMPs by PRR partners, similarly to a typical viral PTI. In this case, one may consider virus derived-dsDNA as possible PAMPs. The viral single-stranded DNA form begomoviruses replicates via double-stranded DNA intermediates that are transcribed in the nucleus of plant-infected cells. NSP binds to the nascent viral DNA and facilitates its movement to the cytoplasm and acts in concert with the classical MP to transport the viral DNA to the adjacent, uninfected cells. Activation of NIK1 in incompatible interactions promotes phosphorylation and subsequent translocation of RPL10 to the nucleus, where it interacts with LIMYB to fully repress the expression of RP genes, leading to global translation suppression, which also impairs viral mRNA (vmRNA) translation. In begomovirus-host compatible interactions, NSP binds to NIK1 and suppresses its activity. In any case, RNA or DNA viruses, a successful infection implicates in accumulation of virus effectors (for example, CP from PPV and NSP from begomoviruses) to suppress PTI, leading to disease. In resistant genotypes, however, the resistance genes specifically recognize, directly or indirectly, the viral effectors, called avirulence (Avr) factors, activating ETI and conferring resistance. Adapted from Machado et al. (2015).

An additional recently uncovered virus-specific defense mechanism relies on suppression of host translation mediated by the transmembrane immune receptor NUCLEAR SHUTTLE PROTEIN-INTERACTING KINASE 1 (NIK1), which was first identified as a virulence target of begomovirus NSP (**Figure 2**). Activation of NIK1-mediated antiviral signaling leads to translocation of the ribosomal protein L10 (RPL10) to the nucleus, where it interacts with L10-INTERACTING MYB DOMAIN-CONTAINING PROTEIN (LIMYB) to fully repress expression of translational machinery-related genes and global host translation. Begomovirus mRNAs are unable to escape this translational regulatory mechanism of plant cells and hence are not efficiently translated, which compromises infection upon activation of NIK1-mediated defense. Although the NIK1-mediated defense response is remarkably dissimilar from the PTI response, structural components and activation of the NIK1 immune receptor as well as its interaction with virus infection exhibit features reminiscent of the plant innate immunity mechanism.

This review focuses on the concepts of viral ETI and viral PTI, describing antiviral immune receptors and co-receptors involved in antiviral innate immunity in plants. Furthermore, we describe NIK1-mediated antiviral signaling, a newly discovered layer of antiviral defense, which is specific to plant DNA viruses and relies on transmembrane receptor-mediated translational suppression for defense. This latter level of antiviral defense is discussed within the context of the innate immune system.

## Effector-Triggered Immunity in Antiviral Defense: R Gene-Mediated Responses to Virus Infection

Activation of ETI, involving strain-specific recognition of a virus-encoded effector through direct or indirect interaction with a corresponding resistance gene (R gene) product, can lead to the hypersensitive reaction (HR). HR is considered a resistance response against several different pathogens that, to the some extent, occurs through similar mechanisms. Similar to non-viral infections, the HR response during viral infection is initiated by direct or indirect Avr-R interactions and is frequently associated with accumulation of SA in both infected and non-infected tissues (Culver and Padmanabhan, 2007; Carr et al., 2010; Pallas and García, 2011; Mandadi and Scholthof, 2012). HR is also associated with perturbation in Ca^++^ homeostasis, membrane integrity and activation of caspase-like proteases, such as the vacuolar processing enzyme that is considered an executioner of cell death during HR (Mur et al., 2008). Although cell death is often associated with HR-mediated resistance, HR may be uncoupled from resistance, an interpretation that arises from compelling biochemical and genetic studies of *Potato virus X* (PVX), *Tomato bushy stunt virus* (TBSV), *Cauliflower mosaic virus* (CaMV) and *Tomato mosaic virus* (ToMV; Bendahmane et al., 1999; Chu et al., 2000; Cole et al., 2001; Ishibashi et al., 2007, 2009). For instance, the tomato resistance protein Tm-1 relays resistance against ToMV by inactivating the ToMV replicase protein without eliciting HR-associated cell death (Ishibashi et al., 2007, 2009).

As for non-viral pathogens, most plant antiviral R genes encode NB-LRR [nucleotide-binding-leucine-rich repeat (LRR)] proteins that mediate resistance via specific (direct or indirect) recognition of a virus Avr factor (Win et al., 2012) (**Table 1**). Based on their variable N-terminal domain, these plant NB-LRR proteins are further classified into coiled-coil (CC)-NB-LRR or Toll/interleukin 1 receptor-like (TIR)-NB-LRR protein families (Bonardi et al., 2012). Most of the known antiviral R proteins are CC-NB-LRR-like, whereas only a small number belong to the TIR-NB-LRR class (Zvereva and Pooggin, 2012; de Ronde et al., 2014). Recognition of effectors by R proteins may occur through direct ligand-receptor interactions (gene-for-gene model; Flor, 1971) or through indirect interactions (Guard Model; Jones and Dangl, 2006; Oßwald et al., 2014). In the Guard Model, the resistance protein guards a target host protein, the guardee, and perceives alterations in this target protein upon interaction with the pathogen effectors. Therefore, the modification of the guardee by the effector causes activation of the R protein to initiate a resistance response. Implicit in the Guard Model is the notion that the guarded effector target is indispensable for the virulence function of the effector protein in the absence of the cognate R protein (Dangl and Jones, 2001; Jones and Dangl, 2006). Alternatively, in the Decoy Model, a decoy (effector target mimic) evolved to act as a molecular sensor to only detect a pathogen without having any other role in the basic cellular machinery of the host (Van der Hoorn and Kamoun, 2008). Therefore, effector alteration of the decoy triggers innate immunity in plants that carry the cognate R protein but does not result in enhanced pathogen fitness in plants that lack the R protein.

**Table 1:** Plant antiviral NB-LRR resistance genes and the cognate avirulence determinants.

Gene	Plant	R protein signature	Virus	Avr factor	Reference
*N*	*Nicotiana glutinosa*	TIR-NB-LRR	Tobacco mosaic virus (TMV)	Replicase	Whitham et al., 1994; Padgett et al., 1997
*Rx1*	*Solanum tuberosum*	CC-NB-LRR	Potato virus *X* (PVX)	Coat Protein	Bendahmane et al., 1999
*Rx2*	*S. tuberosum*	CC-NB-LRR	PVX	Coat Protein	Bendahmane et al., 2000
*HRT*	*Arabidopsis thaliana* ecotype Dijon-17	CC-NB-LRR	Turnip crinkle virus (TCV)	Coat Protein	Cooley et al., 2000; Ren et al., 2000
*RCY1*	*A. thaliana* ecotype C24	CC-NB-LRR	Cucumber mosaic virus strain y	Coat Protein	Takahashi et al., 2001, 2002
*Sw-5*	Solanum peruvianum	SD-CC-NB-LRR	Tomato spotted wilt virus	Movement protein (NS)	Brommonschenkel et al., 2000; Spassova et al., 2001; Hallwass et al., 2014; Peiro et al., 2014
*Y-1*	*S. tuberosum*	TIR-NB-LRR	Potato virus *Y*	?	Vidal et al., 2002
*Tm-22*	*Solanum lycopersicum*	CC-NB-LRR	Tomato mosaic virus (ToMV)	Movement protein	Lanfermeijer et al., 2003
*BcTuR3*	*Brassica campestris*	TIR-NB-LRR	Turnip mosaic virus	?	Ma et al., 2010
*Rsv1*	*Glycine max*	CC-NB-LRR	Soybean mosaic virus	P3 + HC-Pro	Hayes et al., 2004; Wen et al., 2013
*Pv1*	*Cucumis melo*	TIR-NB-LRR	Papaya ringspot virus	?	Anagnostou et al., 2000
Pv2	*Cucumis melo*	TIR-NB-LRR	Papaya ringspot virus	?	Brotman et al., 2013
*Cv* (locus)	*Poncirus trifoliata*	CC-NB-LRR	Citrus tristeza virus	?	Yang et al., 2003
CYR1	*Vigna mungo*	CC-NB-LRR	Mungbean yellow mosaic virus	Coat Protein	Maiti et al., 2012
*Pvr4*	*Capsicum annuum*	CC-NB-LRR	Potato virus *Y* Pepper mottle virus	RNA-dependent RNA polymerase (NIb)	Kim et al., 2015, 2016
*Tsw*	*Capsicum chinense*	CC-NB-LRR	Tomato spotted wilt virus	NSs RNA silencing suppressor	Margaria et al., 2007; Ronde et al., 2013, 2014; Kim et al., 2016

*Avr, avirulence; CC, coiled- coil; NB, nucleotide binding; LRR, leucine-rich repeat; SD, solanaceous-specific domain; TIR, Toll/interleukin 1 receptor-like.*

The R signaling cascade in plant–virus interactions consists of rapid activation of MAP kinases and involvement of molecular chaperone complexes controlling R protein stabilization and destabilization (Kadota and Shirasu, 2012; Hoser et al., 2013). Convergence between viral and non-viral ETI is observed at the chaperone protein complex containing HEAT SHOCK PROTEIN 90 (HSP90), SUPPRESSOR OF THE G2 ALLELE OF SKP1 (SGT1) and REQUIRED FOR MLA12 RESISTANCE1 (RAR1). The HSP90/RAR1/SGT1 chaperone complex contributes to the stability and proper folding of R proteins during activation, mediating downstream MAP kinase activation, changes in defense gene expression and hormone levels (Liu et al., 2004; Dodds and Rathjen, 2010). Examples of R proteins against viruses that use the HSP90/RAR1/SGT1signaling module to mediate antiviral resistance are the N protein and Rx protein, which confer resistance against Tobacco mosaic virus (TMV) and PVX, respectively (**Table 1**) (Liu et al., 2004; Botër et al., 2007). Another functional module comprising ENHANCED DISEASE SUSCEPTIBILITY1 (EDS1; Aarts et al., 1998; Falk et al., 1999), PHYTOALEXIN DEFICIENT4 (PAD4; Feys et al., 2001, 2005) and SENESCENCE-ASSOCIATED GENE101 (SAG101) mediates HR against viral and non-viral pathogens in a similar manner. In Arabidopsis, the EDS1/PAD4/SAG101 complex regulates HRT-mediated resistance against *Turnip crinkle virus* (TCV; **Table 1**) (Zhu et al., 2011). The HRT-mediated resistance also requires a functional SA-mediated signaling pathway (Chandra-Shekara et al., 2004). Disruption of SA signaling compromises HRT-mediated resistance without affecting HRT-mediated HR, providing further evidence that HR and resistance, albeit closely related, are unlinked processes. Therefore, virus-triggered ETI responses also involve functional SGT1/RAR1/HSP90 (Liu et al., 2004) and EDS1/PAD4/SAG101 (Zhu et al., 2011) protein complexes.

The tobacco *N* gene (for necrotic-type response), which confers resistance against TMV and encodes a TIR-NB-LRR protein, was the first identified R gene (Holmes, 1938; Whitham et al., 1994). TMV is a positive-sense single-stranded RNA virus of 6.3–6.5 kb that encodes at least four proteins (Goelet et al., 1982; Osman and Buck, 1996). They include a 126-kDa replicase (with methyltransferase and RNA helicase domains), which is encoded by the 5′ORF of TMV and is directly translated from genomic RNA; the stop codon of which is read through to give a 183-KDa RNA-dependent RNA polymerase (RDR). The other two viral proteins, a MP and a capsid protein (CP), are expressed from separate subgenomic RNAs. The N resistance protein directly interacts with the helicase domain (the p50 effector) of TMV replicase to trigger resistance (Ueda et al., 2006). In fact, ectopic expression of the C-terminal 50 kDa portion (p50) of the 126 kDa replicase is sufficient to induce HR in tobacco carrying the *N* gene (Erickson et al., 1999). Full resistance to TMV, however, depends on N receptor-interacting protein 1 (NRIP1), which is recruited from chloroplasts to the cytoplasm and nucleus by the p50 effector and interacts directly with the N resistance protein (Caplan et al., 2008). The nuclear localization of the N resistance protein, which has been demonstrated to be critical for N-mediated resistance, is controlled by upstream events of receptor activation (Burch-Smith et al., 2007; Hoser et al., 2013). As a plant NB-LRR, the N protein requires the conserved chaperone complex consisting of HSP90, RAR1 and SGT for proper folding, accumulation and regulation (Liu et al., 2004). The assembly of this chaperone complex with the N protein occurs in the cytoplasm and SGT controls the nucleocytoplasmic partitioning of the immune receptor (Hoser et al., 2013). Upon TMV infection, p50 binds first to the TIR domain and then to the NB and LRR domains of the N protein leading to conformational changes and oligomerization of the immune receptor (Mestre and Baulcombe, 2006). Phosphorylation of SGT1 by an activated SIPK, a tobacco MAPK6 homolog, shifts the balance toward its nuclear distribution and consequently the N receptor complex is distributed to the nucleus (Burch-Smith et al., 2007; Hoser et al., 2013). Within the nucleus, N protein interacts with transcriptional factors (TFs) to modulate the expression of defense-related genes. The SQUAMOSA PROMOTER BINDING PROTEIN (SBP)-domain transcription factor SPL6 is an example of TF that interacts with the N immune receptor and positively regulates a subset of defense genes (Padmanabhan et al., 2013). This association is detected only when the TMV effector, p50, is present in the cell and is required for N-mediated resistance. SPL6 from Arabidopsis also functions in resistance against the bacterial pathogen *Pseudomonas syringae* expressing the AvrRps4 effector, as SPL6 is required for the R protein RPS4-mediated resistance (Padmanabhan et al., 2013). Therefore, the SPL6-mediated modulation of defense gene expression represents another convergent point in R-mediated resistance against both viruses and bacteria.

The *Rx* gene in potato encodes a well-characterized representative of the CC-NB-LRR class of R proteins, which mediates extreme resistance against PVX elicited by the viral CP. PVX is also a monopartite positive-sense single-stranded RNA virus (Huisman et al., 1988). Unlike other disease resistance responses, this extreme resistance is not associated with HR at the site of infection but rather is associated with an early arrest of viral accumulation in single cells (Bendahmane et al., 1999). The Rx protein also associates with the molecular chaperone HSP90 and its signaling proteins SGT1 and RAR1 to modulate the innate immune response in plants (Botër et al., 2007). The cochaperone SGT1 also interferes with the nucleocytoplasmic distribution of Rx protein (Slootweg et al., 2010; Hoser et al., 2014). Accordingly, silencing the cochaperone SGT1 impaired the accumulation of Rx1 protein in the nucleus and Rx distribution exactly mirrored that of ectopic AtSGT1b variants with forced cytoplasmic or nuclear localization. The Rx nucleocytoplasmic partitioning is also controlled by the Rx interacting partner RanGAP2 (Tameling et al., 2010). The Rx N-terminal CC domain interacts intramolecularly with the Rx NB-LRR region and intermolecularly with the Rx cofactor RanGAP2 (Ran GTPase-activating protein 2; Rairdan et al., 2008; Tameling et al., 2010). In fact, the crystal structure of the CC domain of Rx in complex with the Trp-Pro-Pro (WPP) domain of RanGAP2 reveals that the Rx CC domain forms a heterodimer with RanGAP2, which may prevent Rx self-association (Hao et al., 2013). The C-terminus of the LRR domain is thought to be involved in specific recognition of the viral effector, CP, although direct interaction between CP and Rx has not been demonstrated (Bendahmane et al., 1995; Dangl and Jones, 2001; Farnham and Baulcombe, 2006; Candresse et al., 2010). The Rx-interacting protein RanGAP2 controls Rx nucleocytoplasmic distribution and can act as a cytoplasmic retention factor for Rx. CP of PVX is recognized in the cytosol, and signaling is also activated in this compartment. Concentrating Rx in the cytosol via RanGAP2 overexpression enhances resistance signaling, whereas sequestering Rx in the nucleus through interaction with a nuclear-localized version of RanGAP2 inhibits resistance signaling (Slootweg et al., 2010; Tameling et al., 2010). However, nuclear export signal-mediated expulsion of Rx from the nucleus moderately reduced resistance, indicating that the nuclear pool of Rx also functions in immunity. These results demonstrate that both nuclear and cytoplasmic pools of NB-LRR Rx1 are necessary for full immune responses to PVX. Therefore, in both Rx-mediated resistance and N-mediated resistance, the R protein is activated in the cytoplasm, yet full functionality of the Rx and N R proteins depends on their nucleocytoplasmic distribution.

A few dominant resistance genes encoding the non-NB-LRR class of proteins have been characterized; these proteins have been found to function as sensors of virus infection but do not induce typical ETI-like defense responses, such as HR (**Table 2**). One such example is the tomato *Tm-1* gene, which confers dominant resistance to ToMV and contains two conserved domains: an uncharacterized N-terminal region (residues M1–K431) and a TIM-barrel-like C-terminal domain (residues T484–E754; Ishibashi et al., 2012, 2014; Yang et al., 2016). Tm-1 binds to ToMV replication proteins and inhibits ToMV multiplication without inducing a defense response: binding of Tm-1 to ToMV replication proteins inhibits the RNA-dependent RNA replication of ToMV and replication complex assembly on membranes that precedes negative-strand RNA synthesis (Ishibashi and Ishikawa, 2013, 2014). Another recently characterized example of non-NB-LRR R proteins is the sensor proteins Ty-1 and Ty-3, which confer resistance to *Tomato yellow Leaf Curl Virus* (TYLCV). The *Ty-1* and *Ty-3* genes are allelic and code for an RDR of the RDRc type, which has an atypical DFDGD motif in the catalytic domain (Verlaan et al., 2013). The mechanism of resistance is completely uncoupled from ETI and appears to be linked to the RNA silencing strategy of antiviral defense (Butterbach et al., 2014). Accordingly, *Ty-1/Ty-3* plants display enhanced siRNA levels that coincide with hypermethylation of the TYLCV V1 (CP) promoter, indicating that *Ty-1*-based resistance against TYLCV involves enhanced transcriptional gene silencing.

**Table 2:** Plant antiviral non-NB-LRR resistance genes and the cognate avirulence determinants.

Gene	Plant	R protein signature	Virus	Avr determinant?	Reference
*JAX1*	*Arabidopsis thaliana*	Jacalin-like [lectin gene]	Broad resistance against potexvirus	?	Yamaji et al., 2012
*RTM1*	*Arabidopsis thaliana*	Jacalin-like	Tobacco etch virus	Coat Protein	Chisholm et al., 2000
*RTM2*	*Arabidopsis thaliana*	Jacalin-like	Plum pox virus	Coat Protein	Whitham et al., 2000; Decroocq et al., 2009
*Ty-1, Ty-3*	*Solanum chilense*	RDR	Tomato yellow leaf curl virus	?	Verlaan et al., 2013; Butterbach et al., 2014
*Tm-1*	*Solanum hirsutum*	TIM-barrel-like domain protein	ToMV	Replicase, Helicase domain	Ishibashi et al., 2007; Kato et al., 2013

*Avr, avirulence; RDR, RNA-dependent RNA polymerase.*

In summary, most antiviral dominant resistance genes so far characterized encode typical NB-LRR R proteins (**Table 1**), which specifically recognize viral effectors or Avr factors and utilize signaling modules such as SGT1/RAR1/HSP90 and EDS1/PAD4/SAG101 complexes to mediate resistance responses, similar to non-viral pathogens. Therefore, plants appear to have evolved strategies and signaling modules to defend themselves against a large spectrum of pathogen types, such as bacteria, viruses and fungi. This interpretation allows us to integrate some aspects of the antiviral immune concepts into the typical bacterial and fungal immunity models to classify viral effectors and viral ETI.

## PAMP-Triggered Immunity in Antiviral Defenses: Co-Receptors Pave the Way

Plant innate defense responses are also activated upon perception of conserved PAMPs, which are pathogen-derived conserved motifs. In addition, endogenous molecules released by the host during pathogenic attack or wounding, which are known as DAMPs, can also elicit plant defense (Zipfel, 2014). Detection of different PAMPs/DAMPs by the corresponding PRRs at the plasma membrane activates signaling cascades, leading to transcriptional and physiological changes in host cells that prevent pathogen infection and establish PTI (Jones and Dangl, 2006; Macho and Zipfel, 2014; Bartels and Boller, 2015). In plants, PRRs are represented by RLKs and RLPs located at the cell surface, both of which require a co-receptor to form an active complex and initiate signaling (Machado et al., 2015). The best characterized co-receptors for PRR are members of LRR subfamily II of the RLK superfamily (LRRII-RLK subfamily). This family is represented by 13 members in the Arabidopsis genome, which can be divided into three closely related clusters: one representing five SOMATIC EMBRYOGENESIS RECEPTOR KINASES (SERK1-5), a cluster of LRR-RLKs of unknown function and a cluster of NUCLEAR-SHUTTLE PROTEIN-INTERACTING KINASES (NIK1-3; Zhang et al., 2006; Sakamoto et al., 2012). Among SERKs, SERK3, which is also termed BRASSINOSTEROID INSENSITIVE 1 (BRI1)-ASSOCIATED KINASE 1 (BAK1), is the most well-characterized subfamily member. SERK3 functions as a co-receptor of several PRRs, such as FLAGELLIN SENSING 2 (FLS2), ELONGATION FACTOR-thermo unstable (EF-Tu) receptor (EFR) or PEP1 receptor 1 (PEPR1), which perceive specific PAMPs/DAMPs and trigger or amplify bacterial/fungal PTI (Chinchilla et al., 2007; Heese et al., 2007; Roux et al., 2011; Wang et al., 2014). Upon PAMP perception, FLS2 and EFR form a ligand-induced complex with BAK1, which leads to rapid phosphorylation of both proteins (Chinchilla et al., 2007; Heese et al., 2007; Roux et al., 2011; Sun et al., 2013) and activation of immune responses, including production of ROS by the NADPH oxidase RBOHD, activation of the mitogen-activated protein kinase (MAPK) cascade, transcriptional reprogramming of defense genes and immunity to pathogens (Kadota et al., 2014; Li et al., 2014; Macho and Zipfel, 2014).

The mechanism of PTI in virus–host interactions is well characterized in animals. One of the best studied PRRs in mammals, Toll-like receptors (TLR), have important roles in antiviral defense via recognition of a different range of MAMPs, such viral RNA and DNA (Song and Lee, 2012). In contrast, the PTI pathway in plants remains unclear with regard to resistance against viruses, although studies describing an association of PTI in antiviral immunity have been recently reported (Yang et al., 2010; Kørner et al., 2013; Nicaise, 2014; Machado et al., 2015; Nicaise and Candresse, 2016; Niehl et al., 2016). Indeed, a complex set of typical PTI responses is induced in plants upon virus infection, including SA accumulation, ROS production, ion fluxes, defense gene (e.g., PR-1) activation, and callose deposition (for a review, see Nicaise, 2014). The PRR co-receptors BAK1 or BAK1-LIKE1 (BKK1) are required for antiviral immunity in Arabidopsis, and loss-of-function mutations in *BAK1* and *BKK1* result in enhanced susceptibility to TCV infection (Yang et al., 2010). Consistently, Arabidopsis *bak-1* mutants show increased susceptibility to three different RNA viruses, and crude extracts of virus-infected leaf tissues induce typical PTI responses in a BAK1-dependent manner (Kørner et al., 2013). The Arabidopsis double mutant *bak1-5 bkk1* displays increased viral accumulation when inoculated with *Plum pox virus* (PPV; Nicaise and Candresse, 2016). Furthermore, MAPK4, a negative regulator of plant PTI signaling, suppresses soybean defense against *Bean pod mottle virus* (BPMV; Liu et al., 2011), and chitosan, a deacetylated chitin derivative elicitor, is able to stimulate the plant immune response against viruses (Iriti and Varoti, 2014).

The current data suggest that viral components can act as PAMPs but do not eliminate the possibility that DAMPs produced in response to virus can potentially elicit PTI-based antiviral responses in plants. Recently, double-stranded RNA (dsRNA) and virus-derived dsRNA have been shown to function as viral PAMPs in Arabidopsis and to induce the PTI pathway (Niehl et al., 2016). Indeed, application of dsRNA to Arabidopsis leaf disks resulted in the induction of typical PTI responses, including MAPK activation, ethylene synthesis and defense gene expression. Furthermore, dsRNA treatment confers protection against viruses because plants inoculated with the synthetic dsRNA analog polyinosinic–polycytidylic acid, poly(I:C) together with *Oilseed rape mosaic virus* (ORMV) showed significantly reduced viral accumulation in treated leaves. Interestingly, dsRNA-mediated PTI appears to be independent of the RNA silencing pathway but does involve the co-receptor kinase SERK1. These findings relate membrane-associated signaling events with dsRNA-mediated PTI in plants (Niehl et al., 2016). Although plasma membrane-localized co-receptors of PRRs, such as BAK1, BKK1 and SERK1, have been shown to be involved in viral PTI, it remains to be determined how intracellular pathogens, which deliver PAMPs intracellularly, are perceived extracellularly.

The PTI pathway also contributes to antiviral immunity against PPV in Arabidopsis (Nicaise and Candresse, 2016). As a counteraction strategy, the CP from PPV appears to act as a PTI suppressor, impairing early immune responses such as the oxidative burst and enhanced expression of PTI-associated marker genes *in planta* during infection (Nicaise and Candresse, 2016). Therefore, PPV CP displays a virulence function that acts at the PTI level and antagonizes the Avr functions of many viral recognized by antiviral R proteins during elicitation of ETI (**Table 1**). These observations suggest that plant viruses also fit into the zigzag model of co-evolving pathogenic virulence strategies and plant defense responses that shape the two-branched innate immune system (Jones and Dangl, 2006).

Collectively, these data suggest the existence of PTI signaling mechanism targeting plant viruses and may represent a conserved process between plants and animals. Identification of PRR-mediated pathways as well as characterization of nucleic acid-sensing PRRs will shed light on the mechanism by which PTI is elicited in plants and its role in antiviral resistance.

## Transmembrane Receptor-Mediated Translational Suppression in Antiviral Immunity: Unique and Shared PTI-Like Features of the NIK1-Mediated Antiviral Response

The transmembrane receptor NIK was first identified as a virulence target of Nuclear Shuttle Protein (NSP) from *Begomovirus*, the largest genus of the *Geminiviridae* family (Fontes et al., 2004; Mariano et al., 2004). Similar to the PTI co-receptors BAK1 and SERK1, NIK receptors (NIK1, NIK2 and NIK3) belong to the LRRII-RLK subfamily and are involved in plant defenses against viruses (Fontes et al., 2004). Nevertheless, the mechanism by which NIK1 transduces the antiviral signal is completely different from the typical PTI signaling mediated by BAK1 or SERK1 and PRRs. Nonetheless, some similarities between these transduction pathways with regard to receptor activation, suppression and association with ETI have been observed (Machado et al., 2015, **Figure 2**).

NUCLEAR SHUTTLE PROTEIN-INTERACTING KINASE 1-mediated antiviral signaling is activated upon perception of begomovirus infection, which leads to phosphorylation of the NIK1 kinase at key threonine residues at positions 468 and 474 (Santos et al., 2009; Zorzatto et al., 2015). Thr-468 and Thr-474 are located within the conserved activation loop and align to the same positions as conserved BAK1 residues Thr-449 and Thr-455 and SERK1 residues Thr-462 and Thr-468, which are intramolecular targets for BAK1 and SERK1 kinase activation (Shah et al., 2001; Wang et al., 2005, 2008; Yun et al., 2009). Phosphorylation of the functional analogs NIK1 Thr-474, SERK1 Thr-468 and BAK1 Thr-455 is essential for receptor/co-receptor signaling, which may underscore a similar mechanism for activation (Shah et al., 2001; Wang et al., 2008; Santos et al., 2009; Yun et al., 2009; Brustolini et al., 2015). Nevertheless, unlike BAK1 or SERK1, phosphorylation at NIK1 Thr-474 leads to phosphorylation at Thr-469, which has an inhibitory effect, thereby providing a mechanism by which NIK1 modulates the extent of auto- and substrate phosphorylation. Although NIK1 is activated upon perception of virus infection, the molecular bases of such elicitation are unknown. Indeed, there is a complete lack of information with respect to the nature and identity of possible ligands or mechanisms that trigger or stabilize NIK1 dimerization or multimerization with receptors. Because viruses are intracellular pathogens and may not have access to the apoplast, it remains to be determined how the NIK1 extracellular domain, which is expected to drive ligand-dependent oligomerization of receptors and co-receptors, senses viruses intracellularly. Possible ligands that could perform this function are DAMPs, which would be secreted by plant cells upon virus perception. Alternatively, viral nucleic acid-derived PAMPs could intracellularly activate NIK1 kinase, a mechanism that would resemble virus-derived dsRNA-mediated activation of mammalian intracellular protein kinase R (PKR; Balachandran et al., 2000). Virus-derived nucleic acid PAMPs could also activate NIK1-associated nucleic acid-sensing PRRs in endosomes derived from receptor internalization via endocytic pathways. In plant cells, the PRRs FLS2, ERR and PEPR have been shown to be internalized in a clathrin-dependent manner. Endocytosis requires the co-receptor BAK1 and depends on receptor activation (Mbengue et al., 2016). In a similar manner, the Avr factor Avr4 induces association of Cf-4 RLP with BAK1 to initiate receptor endocytosis and plant immunity (Postma et al., 2016).

In general, ligand-dependent phosphorylation and activation of RLKs require homo or heterodimerization of the receptors. In the case of BAK1 and SERK1, compelling evidence has revealed that they function primarily as co-receptors for receptor signaling not only in defense but also in development (Ma et al., 2016). As a member of the LRRII-RLK subfamily sharing conserved structural organization and biochemical activation properties with SERKs, NIK1 may also function as a co-receptor in immune active complexes. However, NIK1-containing antiviral signaling complexes have not been isolated, and a receptor partner for NIK1 has yet to be identified.

Begomovirus NSP binds *in vitro* and *in vivo* with the kinase domain of NIK1 to suppress NIK1 activity (Fontes et al., 2004; Brustolini et al., 2015). The NSP binding site corresponds to an 80-amino acid stretch (positions 422–502) of NIK1 that encompasses the putative Ser/Thr kinase active site (subdomain VIb–HrDvKssNxLLD) and the activation loop (subdomain VII–DFGAk/rx, plus subdomain VIII–GtxGyiaPEY; Fontes et al., 2004). Binding of NSP to the kinase domain promotes steric constraints that impair intermolecular phosphorylation at Thr-474 within the A-loop of NIK1, thereby suppressing its kinase activity. The NSP-mediated suppression of NIK1 kinase prevents activation of the NIK-mediated pathway and hence enhances the pathogenicity of begomoviruses in their hosts (Santos et al., 2009, 2010). In addition to acting as a virulence factor suppressing NIK1-mediated antiviral signaling, NSP from the begomovirus *Bean dwarf mosaic virus* (BDMV) has been demonstrated to function as an Avr gene and elicit HR in *Phaseolus vulgaris* (Garrido-Ramirez and Gilbertson, 1998). According to the zigzag evolutionary model of plant innate immunity (Jones and Dangl, 2006), the involvement and activation of ETI in plant-virus interactions (NSP in resistant bean genotypes) is conceptually associated with successful PTI inhibition (NIK1 signaling) by a viral effector (NSP). This interpretation further substantiates the notion that NIK1-mediated antiviral signaling shows features of PTI-like mechanisms.

Despite similarities in the activation and suppression mechanisms of PTI and NIK1-mediated antiviral signaling, the downstream events of NIK1 activation are quite distinct from the typical PTI response. In fact, activation of NIK1 signaling by constitutive or inducible expression of the gain-of-function T474D NIK1 mutant, which is not inhibited by viral NSP, results in a massive down-regulation of translation machinery-related genes, suppression of host global translation and enhanced broad-spectrum tolerance to begomoviruses in Arabidopsis and tomato (Brustolini et al., 2015; Zorzatto et al., 2015). T474D-mediated suppression of global translation is associated with a decrease in host and viral mRNA in actively translating polysomes. Therefore, begomovirus is not capable of sustaining high levels of viral mRNA translation in T474D-expressing lines, indicating that suppression of global protein synthesis may effectively protect plant cells against DNA viruses.

Progress toward deciphering the mechanism of the translational control branch of NIK1 signaling includes identification of the downstream effectors, RPL10 and LIMYB (Rocha et al., 2008; Zorzatto et al., 2015). RPL10 was isolated based on its capacity to interact with NIK1 and was genetically and biochemically linked to the NIK1-mediated signaling pathway (Carvalho et al., 2008; Rocha et al., 2008). Consistent with a role for RPL10 in antiviral defense, loss of *RPL10* function recapitulates the *nik1* enhanced susceptibility phenotype to begomovirus infection, as *rpl10* knockout lines develop severe symptoms similar to those of *nik1* and display a similar infection rate (Carvalho et al., 2008; Rocha et al., 2008). NIK1 activation mediates RPL10 phosphorylation and consequent translocation of the RP from the cytoplasm to the nucleus. The regulated nucleocytoplasmic shuttling of RPL10 depends on NIK1 kinase activity and on the phosphorylation status of the RP (Carvalho et al., 2008). Mutations that impact NIK1 activity similarly affect the capacity of NIK1 to mediate translocation of RPL10 to the nucleus and to transduce an antiviral signal. In the nucleus, RPL10 interacts with LIMYB to form a transcription-repressing complex that specifically down-regulates expression of translational machinery-related genes, such as RP genes. This down-regulation of RP genes results in global suppression of host translation and enhanced tolerance to begomoviruses. Expression of the gain-of-function T474D mutant also results in repression of the same set of LIMYB-regulated RP genes, but T474D requires the function of LIMYB for RP repression. In addition, loss of LIMYB function releases the repression of translation-related genes and increases susceptibility to *Cabbage leaf curl virus* (CaLCuV) infection (Zorzatto et al., 2015). Collectively, these results provide both genetic and biochemical evidence that LIMYB functions as a downstream component of the NIK1-mediated signaling pathway linking NIK1 activation to global translation suppression and tolerance to begomoviruses.

Although NIK1 is structurally similar to SERKs, the mechanism of NIK1-mediated antiviral defense is distinct from that of BAK1-mediated PTI. The current model for NIK1-mediated antiviral signaling states that, in response to virus infection, NIK1 undergoes homo- or heterodimerization to promote phosphorylation of the activation loop (**Figure 2**). Activated NIK1 mediates phosphorylation and consequent translocation of RPL10 to the nucleus, where it interacts with LIMYB to fully repress RP gene expression. Prolonged down-regulation of RP gene expression leads to suppression of global host translation. DNA viruses, such as begomoviruses, cannot escape this translational regulatory mechanism of plant cells, and viral mRNAs are not translated efficiently, thereby compromising infection. NSP acts as a virulence factor and suppresses the kinase activity of NIK1 to overcome the NIK1-mediated immune response. NSP from the begomovirus BDMV has also been shown to function as an Avr factor that activates typical ETI responses in resistant bean genotypes. Therefore, NSP may link the suppression of NIK1 signaling with activation of ETI responses in accordance with the zigzag evolutionary model of plant innate immunity, although the NIK1-mediated antiviral signaling may represent a new evolved branch of plant antiviral immunity, which relies on suppression of translation for defense.

## Conclusion

Innate immunity against plant viruses and its underlying mechanisms have attracted the attention of breeders and scientists. Accordingly, there is a growing list of R genes against viruses, and our knowledge regarding the mechanisms of R gene-mediated defenses has advanced considerably over the last decade. However, in comparison with R genes against non-viral pathogens, the number of well-studied examples of antiviral R genes is still limited with respect to an understanding of the level of specialization of dominant resistance against viruses and the boundaries of features shared with non-viral ETI. Even more limited is our understanding of viral PTI in plants. Recent studies have provided insights into plant viral PTI. For example, it is now known that several components of bacterial and fungal PTI participate also in viral PTI. These include the co-receptor SERKs, BAK1 and SERK1, and the MAPK4 negative regulator, in addition to common effects of non-viral PTI that are also elicited during virus infection. Nevertheless, our knowledge about the dynamics between the virulence strategy of viruses and the plant immune system is still rudimentary, and several steps in the mechanism of antiviral innate immunity are still unknown. Indeed, although non-self RNA motifs appear to function as PAMPs from RNA viruses, we do not know the identities of virus-derived PAMPs or plant-derived DAMPs that would induce antiviral PTI. The repertoire of viral suppressors of PTI is limited to the CP from PPV and perhaps to NSP from begomoviruses. Furthermore, antiviral PRRs have not been identified, and mechanisms by which intracellular pathogens that have no access to the apoplast are sensed extracellularly are unknown. A better understanding of the repertoire of virus effectors (Avr factor) and NB-LRR host targets (R proteins) and their mode of action in activating ETI and/or suppressing PTI will help to define the evolutionary pressure acting upon the host and viruses and to determine how to deploy the immune system for more efficient control of virus infection. We also need to define NIK1-mediated suppression of translation as a general or virus-specific antiviral strategy in plants. To date, a sustained NIK1 pathway has been shown to be effective against begomoviruses, one of the largest groups of plant DNA viruses, which cannot circumvent the regulatory mechanism of host translation. In this regard, the intrinsic capacity of agronomically relevant crops to withstand the deleterious effects of suppression of global translation must be considered as a relevant agronomic trait if we are to use the translational control branch of NIK1-mediated antiviral signaling for crop protection against begomoviruses.

## Author Contributions

BG and IC wrote the first draft of the manuscript; JM wrote the ETI section; AS edited the manuscript and EF edited the manuscript.

## Conflict of Interest Statement

The authors declare that the research was conducted in the absence of any commercial or financial relationships that could be construed as a potential conflict of interest.
